# Cecal Metabolome Profiles of Turkey Poults in Response to *Salmonella* Heidelberg Challenge with or Without Turkey-Derived *Lactobacillus* Probiotic and *Trans*-Cinnamaldehyde

**DOI:** 10.3390/ani15142016

**Published:** 2025-07-08

**Authors:** Grace Dewi, Ranjith Ramanathan, Anup Kollanoor Johny

**Affiliations:** 1 Department of Animal Science, University of Minnesota, Saint Paul, MN 55108, USA; dewix001@umn.edu; 2 Department of Animal and Food Sciences, Oklahoma State University, Stillwater, OK 74078, USA; ranjith.ramanathan@okstate.edu

**Keywords:** pre-harvest, metabolomics, *Salmonella*, poultry, *Trans*-Cinnamaldehyde, *Lactobacillus*

## Abstract

*Salmonella* colonization in the gastrointestinal tract of turkeys presents a risk to the safety of products derived from them. *Lactobacillus*-based probiotics and a plant-derived compound, *trans*-cinnamaldehyde, have previously been found to be effective in reducing multidrug-resistant *Salmonella enterica* subsp. *enterica* serovar Heidelberg (*S.* Heidelberg) in turkey poults. However, the effect of the challenge and the application of the treatments on the cecal metabolome has yet to be elucidated. Thus, the objective of the present study was to characterize alterations in the metabolic profiles of cecal contents collected from poults following *S.* Heidelberg challenge and treatment with *Lactobacillus salivarius* UMNPBX2 and *L. ingluviei* UMNPBX19 (LB), *trans*-cinnamaldehyde (TC), or a combination of both (CO) using untargeted gas chromatography–mass spectrometry (GC-MS). Poults in the challenged control (PC) group had the most distinct and convergent metabolome profiles, with the most pronounced disparity observed compared to the unchallenged control (NC), indicating the effect of the *S.* Heidelberg challenge. Perturbations in metabolites in the primary bile acid biosynthesis, pentose and glucuronate interconversions, and steroid biosynthesis were the most prominent. The greater abundance of metabolites, such as primary bile acids and sugars, in the PC group may be associated with *S.* Heidelberg colonization or potential shifts in microbiota. The treatments yielded varying effects on the metabolome profiles, with the TC and CO groups exhibiting the closest similarity, although TC was more similar to NC. The findings revealed alterations to ceca-associated metabolites, which are likely a response to the *S.* Heidelberg challenge and the application of the TC and LB treatments. Additional studies are needed to validate the possible causal relationship between the observed shifts. Gaining insight into the alterations to the metabolic microenvironment in the avian cecum will help elucidate the mechanisms by which they facilitate *Salmonella* persistence. Understanding these relationships can aid in designing more effective pre-harvest *Salmonella* control strategies and enhancing the efficacy of interventions within the flock.

## Simple Summary

*Salmonella* is a bacterium that can live in the intestines of turkeys and contaminate turkey products, causing human illness. In this project, we aimed to test multiple natural antimicrobial options for reducing *Salmonella* in young turkeys and to study their effects on the intestinal environment. We tested two strains of probiotics and one plant compound, known as *trans*-cinnamaldehyde, which is found in cinnamon, to evaluate whether they could alter the gut environment to make it unfavorable for *Salmonella* colonization. We also examined how the treatments affected the small metabolites within the turkey guts, which can give hints about gut health. Our findings indicated that *Salmonella* infection changed the gut condition, but it was restored to near normal through the natural options, particularly *trans*-cinnamaldehyde. This suggests that these natural antimicrobials may not only suppress undesirable bacteria but also support gut health in turkeys. By understanding how these treatments work, farmers can utilize safer, more natural options to make turkey products safer for everyone, minimizing the risk of foodborne illness.

## 1. Introduction

The consumption of contaminated turkey is the seventh largest contributor to non-typhoidal *Salmonella* infections in the United States, accounting for 5.9% of the infections in 2020 [1]. Reducing infections caused by *Salmonella* is among the nation’s health priorities outlined in the Healthy People 2030 goals [2]. Although the U.S. Department of Agriculture’s (USDA) Food Safety and Inspection Service reported a decrease in *Salmonella* prevalence on poultry products over the past two decades, a corresponding reduction in the incidence of illnesses caused by *Salmonella* has not been observed. Federal data demonstrated that the incidence rate per 100,000 increased from 14 in 2000 to 17 in 2019. Although a sharp decrease was observed in 2020 (13.34 per 100,000), the rate returned to 14 by 2021 [3]. The lack of meaningful progress in reducing salmonellosis, particularly that related to poultry consumption, has prompted USDA officials to announce their plans to reinvigorate studies investigating innovative control strategies to decrease *Salmonella* illness associated with poultry products. One of their primary focuses includes encouraging pre-harvest *Salmonella* interventions, which are among the critical components of their proposed approach [4].

*Salmonella enterica* serovar Heidelberg (*S.* Heidelberg), a pathogen first identified in Heidelberg, Germany, in 1933, has been associated with outbreaks linked to poultry and cattle [5,6]. It was the third most common serotype isolated from retail meat and food animals and the fifth most common serotype in humans in 2009 [7]. Isolates from live animals and foods demonstrated resistance to multiple antibiotics, including tetracycline, streptomycin, and kanamycin, with broadening resistance profiles observed among more recent isolates [8,9]. Notably, a high proportion of hospitalizations have been observed in outbreaks involving multidrug-resistant (MDR) *S.* Heidelberg [5,6,10]. However, the proportion of infections caused by *S.* Heidelberg has since decreased with the emergence of serovars such as *S.* Newport, *S.* Infantis, and *S.* Javiana [3]. The use of a live attenuated *S.* Typhimurium vaccine has been associated with a decline in *S.* Heidelberg infections, likely due to the antigenic similarity between *Salmonella* strains in the same O serogroups [11,12]. However, these interventions afforded only partial protection as they do not affect all *Salmonella* within the flocks, potentially due to the diversity in antigens among the serovars [13,14]. An isolated application of a single intervention may be insufficient to control *Salmonella,* as it persists even among vaccinated birds. The combination of interventions may provide better protection and enhance the efficacy of these preventive measures in reducing *Salmonella* persistence within the flock.

Neonatal animals have a higher predisposition to colonization by bacterial pathogens due to the absence of a fully established protective gut microbiota, which is yet to be established in the young. Kim et al. [15] demonstrated this through transfers of adult germ-free mice with the microbiota of neonates or adults. The neonatal microbiota was unsuccessful in preventing the colonization of two bacterial pathogens. A major obstacle in controlling *Salmonella* is its undetected presence in food-producing animals.

The metabolome, comprising the assortment of low-molecular-weight metabolites with molecular weights between 50 and 1500 Da, is important, as sugars, amino acids, and other compounds in the intestinal lumen also serve as signaling molecules by which Gram-negative enteric pathogens determine the appropriate time to express virulence factors [16]. This ensures that the pathogen does not expend unnecessary energy, which may undermine its fitness to contend with the resident microbes in the gastrointestinal tract, especially since some microbes may produce a multitude of compounds that affect their ability to colonize or survive. In this regard, probiotics and plant-derived antimicrobials have succeeded in reducing *Salmonella* in the gastrointestinal tracts of poultry. Both *trans*-cinnamaldehyde and the *Lactobacillus* strains investigated in this study have demonstrated efficacy against *S.* Heidelberg independently [17,18].

However, studies investigating the mode of action of these alternative interventions, either individually or in combination, in the ceca—the site of *Salmonella* colonization—have not been examined from a metabolomic perspective. Studying the dynamic changes in the chemical landscape during infection and treatment could inform the industry about selecting *Salmonella* intervention strategies that are beneficial for producers and turkey health. Thus, the objective of the study was to characterize the changes in the cecal metabolome of turkey poults in response to the *S.* Heidelberg challenge and how these changes are affected by prophylactic supplementation with *Lactobacillus*, *trans*-cinnamaldehyde, or their combination.

## 2. Materials and Methods

### 2.1. Bacterial Strains and Growth Conditions

#### 2.1.1. S. Heidelberg

The *S.* Heidelberg strain used in this study is MDR and was obtained from ground turkey implicated in the 2011 outbreak (Dewi et al., 2022) [17,18]. The isolate was stored at −80 °C and grown in 10 mL trypticase soy broth (TSB; catalog no. C7142, Criterion, Hardy Diagnostics, Santa Maria, CA) for 24 h at 37 °C. To selectively enumerate the strain, resistance to nalidixic acid (NA; Catalogue no. N4382-25G, Sigma-Aldrich, St. Louis, MO, USA) was induced at 50 μg/mL. Bacterial cultures were inoculated in TSB and underwent three successive propagations (24 h at 37 °C), after which the final culture was sedimented by centrifugation (3600× *g* for 15 min at 4 °C). The inoculum was prepared by serially diluting the resuspended culture using phosphate-buffered saline (PBS, pH 7.2). The growth of *Salmonella* was determined by serial dilution and plating on xylose lysine deoxycholate agar (XLD; catalog no. C7322, Criterion, Hardy Diagnostics, Santa Maria, CA, USA) at 37 °C for 24 h.

#### 2.1.2. *Lactobacillus salivarius* (*L. salivarius*) and *Lactobacillus ingluviei* (*L. ingluviei*)

The *Lactobacillus* strains used in this study were obtained from the ileum of commercial turkeys. Frozen stock cultures (−80 °C) of *L. salivarius* UMNPBX2 (NCBI sequence: NZ_PCZH00000000.1) and *L. ingluviei* UMNPBX19 (NCBI sequence: NZ_PCYR00000000.1) were separately grown in de Man–Rogosa–Sharpe (MRS; catalog no. C5932, Criterion, Hardy Diagnostics, Santa Maria, CA) broth under aerobic conditions at 37 °C for 24 h. All *Lactobacillus* cultures were subjected to three consecutive transfers and were enumerated by plating appropriate dilutions on MRS agar and incubating at 37 °C for 48 h. Both strains were grown separately in 500 mL of MRS at 37 °C for 24 h to prepare inoculum for drinking water. After incubation, the broth, containing approximately 9 Log_10_ CFU/mL of *Lactobacillus*, was centrifuged at 10,000 rpm for 20 min at 4 °C (Allegra X-15 benchtop centrifuge, Beckman Coulter Inc., Fullerton, CA, USA). The bacteria were resuspended in 100 mL PBS and added to the drinking water.

### 2.2. Plant-Derived Antimicrobial (PDA)

The *trans*-Cinnamaldehyde (TC; Food Grade, FCC; Catalogue no W228605-1KG-K) used in this study was purchased from Sigma Aldrich (St. Louis, MO, USA). It was resuspended in water and added directly to the drinking water at a concentration of 0.08% (vol/vol).

### 2.3. Salmonella Challenge Study in Turkey Poults

#### 2.3.1. Ethics Statement

The Institutional Animal Care and Use Committee at the University of Minnesota approved the studies conducted in turkey poults (Approval numbers 1701-34538A and 2008-38400A), and the Institutional Biosafety Committee (Approval numbers 1706-34893H and 2006-38232H) approved the use of infectious agents in the experiments.

#### 2.3.2. Experimental Design, Poults, and Housing

**Figure 1:**
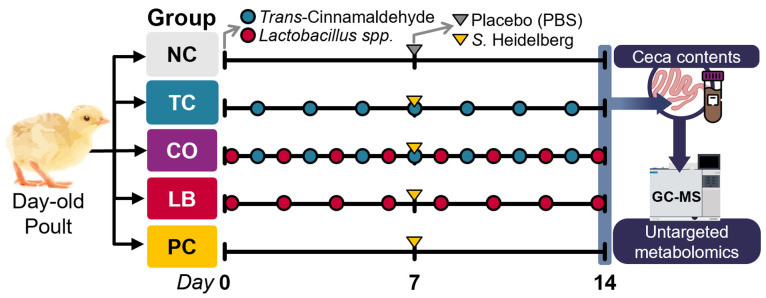
Experimental design. NC—negative control; TC—*trans*-cinnamaldehyde; LB—*Lactobacillus* strains; CO—combination of TC and LB; PC—positive control.

The experimental design and timeline are illustrated in Figure 1. Forty, 1-day-old, straight-run (equal male and female) hybrid converter poults were procured from a commercial turkey hatchery in Minnesota (Select Genetics, Willmar, MN, USA). The poults were housed in the Research Animal Resources’ Biosafety Level 2 Veterinary Isolation Facility at the University of Minnesota. Each containment isolator was equipped with age-appropriate lighting, temperature controls, and sufficient floor space for the turkey poults. The poults were provided *Salmonella*-free ad libitum feed (Famo Feeds Inc., Freeport, MN, USA) and water throughout the study. Feed, fecal, and litter samples were collected in sterile Whirl-Pak bags upon the arrival of the poults. Samples were enriched in 20 mL of selenite cysteine broth (SCB; catalog no. C6921, Criterion, Hardy Diagnostics, Santa Maria, CA, USA) and incubated at 37 °C for 24 h. They were then streaked on XLD plates to determine the presence of inherent *Salmonella*.

Two independent experiments were conducted, and in each, birds were randomly assigned to one of five groups, with eight poults per group. The treatment groups included the TC-only group (TC; 0.08% TC), *Lactobacillus*-only group (LB; 10^9^ CFU/mL of *L. salivarius* and *L. ingluviei*), and combination group (CO; 0.08% TC and 10^9^ CFU/mL of *L. salivarius* and *L. ingluviei*) in 1 gallon of drinking water. The control groups included a negative control (NC; poults neither challenged with *S.* Heidelberg nor supplemented with any intervention) and a positive control (PC; challenged with *S.* Heidelberg without any intervention). *Trans*-cinnamaldehyde and lactobacilli were supplemented on alternate days, thoroughly mixed in drinking water by vigorously shaking the drinkers twice daily. Emulsifying agents were not used to avoid confounding effects and potential reduction in the efficacy of treatments. Upon arrival, *Lactobacillus* was first provided to the LB and CO groups, and *trans*-cinnamaldehyde was supplemented to the TC and CO groups on subsequent days.

On day seven, the poults in the treatment (TC, LB, CO) and PC groups were inoculated with 4.5 log10 CFU of *S.* Heidelberg per bird, delivered by crop gavage. The challenge dose was validated in preliminary experiments. The poults in the NC group received sterile PBS by oral gavage. Treatments were continuously applied until euthanasia by carbon dioxide asphyxiation was performed on day 14. Cecal contents for metabolomics analysis were collected in sterile tubes and immediately stored at −80 °C.

### 2.4. Metabolomics Analysis of Cecal Contents

Cecal contents collected from eight birds in each experimental group were selected at random for metabolomic analysis and metabolite identification at the National Institute of Health West Coast Metabolomics Center at the University of California Davis, CA, USA. Individual cecal samples from each bird were portioned into four sets of 10-milligram aliquots and submitted for quadruplet analysis. Appropriate sample preparation, extraction, and derivatization procedures were followed for metabolite profiling by gas chromatography (GC) and Time-of-Flight (TOF) mass spectrometry (MS).

To extract metabolites, two 3 mm grinder beads and 1000 μL of degassed acetonitrile/isopropanol/water mixture (3:3:2, *v*/*v*/*v*) were added to each sample. The samples were homogenized for 1 min and shaken for 6 min at 4 °C. The homogenates were centrifuged for 2 min at 14,000× *g* and dried under a gentle stream of nitrogen gas before derivatization. The first step of the derivatization involved methoximation with 10 μL of MeOX (40 mg/mL O-methylhydroxylamine hydrochloride) in pyridine. For the second step, 90 μL of trimethylsilylating reagent (MSTFA) was added to increase compound volatilities by exchanging acidic protons [19]. Methods for metabolome profiling were followed as reported by Fiehn and collaborators [20].

### 2.5. Statistical Analysis

All experiments followed a completely randomized design. Data were normalized by systematic error removal by random forest to correct for batch effects or instrument signal drifts [21]. Datasets were then logarithmically (Log_10_) transformed before analysis. Principal coordinate analysis (PCoA) was performed in R (R version 4.3.0, R Core Team, Vienna, Austria) and visualized using the ggplot2 package 3.5.1. Treemap was created in Microsoft Excel. The separation of cecal metabolite profiles across groups was assessed using analysis of similarity (ANOSIM). The proportion of variation contributed by the experimental groups was evaluated using pairwise permutational multivariate analysis of variance (PERMANOVA) on Euclidean distances with 999 permutations. Both tests were performed in R. One-way ANOVA and Fisher’s Least Significant Difference (LSD) post hoc test were performed in R to determine differences across groups (*p* < 0.05). Additionally, non-parametric Kruskal–Wallis tests were conducted in R to assess significance across groups, followed by the Wilcoxon rank sum test for pairwise comparisons between each group (*p* < 0.05). Partial least squares discriminant analysis (PLS-DA), Spearman rank correlations, heatmap, and pathway analysis were performed using MetaboAnalyst 5.0 [22]. Quantitative pathway enrichment and topology analysis were conducted and mapped using the *Gallus gallus* (chicken) KEGG library.

## 3. Results

**Figure 2:**
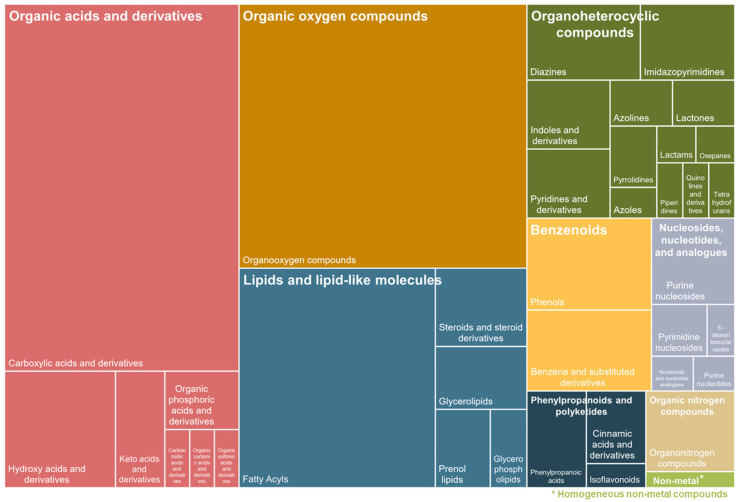
Treemap of annotated metabolites detected through untargeted gas chromatography (GC) Time-of-Flight (TOF) mass spectrometry. Out 895 detected metabolites, 246 were matched to known metabolites. The annotated compounds were further grouped using ClassyFire categories based on their general structural identifiers (Super Class—color) and then by more specific structural features (Class—boxes).

A total of 895 features were detected through untargeted GC-TOF-MS-based metabolomic analysis, of which 246 were matched to known metabolites. The annotated compounds were further grouped using ClassyFire categories (classyfire.wishartlab.com) [23] based on their general structural identifiers (Super Classes) and then by more specific structural features (Class). The results are depicted in Figure 2. Carboxylic acids and their derivatives accounted for 24% of all identified metabolites, followed closely by organooxygen compounds at 22% and fatty acyls at 12%.

**Figure 3:**
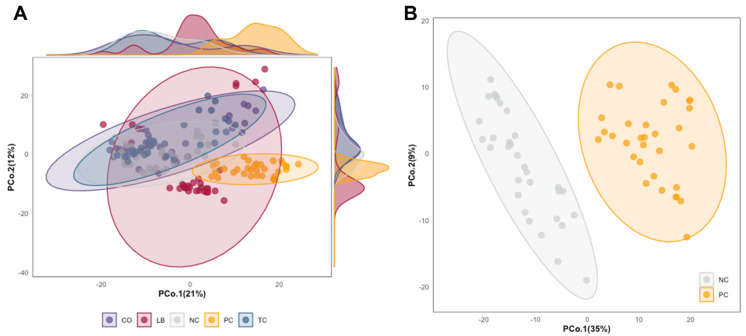
Principal coordinate analysis (PCoA) based on the Euclidean distance metric of log-transformed cecal metabolome data. The ellipses were constructed based on multivariate normal distribution at 95% confidence level. (**A**) PCoA of all experimental groups with density plot along margins. (**B**) PCoA focused on control groups. Abbreviations: NC—negative control; TC—*trans*-cinnamaldehyde; LB—*Lactobacillus* strains; CO—combination of TC and LB; PC—positive control.

The relationship between cecal metabolomic profiles across the experimental groups was visualized using PCoA ordinations based on Euclidean distances (Figure 3). Significant overlap is visible between the NC, TC, and CO groups on the PCoA plot in Figure 3A. Most of the LB samples cluster closely together, but a fourth of the samples were distributed further from the original cluster. By contrast, samples from the PC group were uniformly clustered and positioned further away from the other groups. The separation between the control groups is more prominent when a subset of just the NC and PC is used for analysis, as illustrated in Figure 3B.

This distinction is further supported by the results of both the ANOSIM and PERMANOVA tests, which are summarized in Supplementary Table S1. The greatest effect size (R^2^) and R statistic were observed between PC and NC (R^2^ = 0.305, R statistic = 0.869, *p* = 0.001), indicating that the two groups show the greatest dissimilarity among all groups. In contrast, the smallest values were observed between TC and CO (R^2^ = 0.064, R statistic = 0.153, *p* = 0.001), indicating that the dissimilarities between the two groups do not outweigh the dissimilarities within them, as observed by the overlapping ellipses on the PCoA plot (Figure 3A). The R statistic threshold considered sufficient to indicate a significant difference between the means of two dissimilarities is set at values greater than 0.4 [24]. In addition to NC, the PC group differs significantly from all the treatment groups (R statistic > 0.5). Among the treatment groups, only LB had an R statistic > 0.4 compared to the NC, indicating that the LB group was the treatment group with the greatest separation from the unchallenged control. The NC group showed the least dissimilarity with the TC group. The TC, CO, and LB groups have a decreasing R statistic and effect size compared to the PC group in that order. Likewise, the reverse is observed when compared to the NC group. The CO group showed less dissimilarity with the LB group compared to the NC group, while the LB group had a lower R statistic and R^2^ with TC than the NC group.

**Figure 4:**
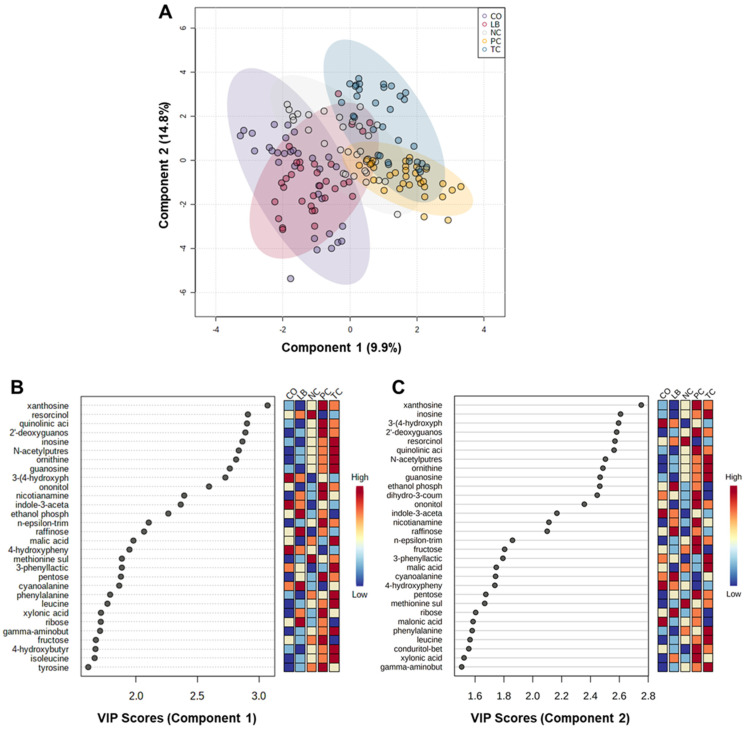
Partial least squares discriminant analysis (PLS-DA). (**A**) PLS-DA score plot comparing the metabolome profiles of all groups. The shaded ellipses indicate the 95% confidence regions. (**B**) Top 30 metabolites ranked based on the PLS-DA variable importance in projection (VIP) score for component 1. (**C**) Top 30 metabolites ranked based on the PLS-DA VIP score for component 2. Abbreviations: NC—negative control; TC—*trans*-cinnamaldehyde; LB—*Lactobacillus* strains; CO—combination of TC and LB; PC—positive control.

PLS-DA, a supervised multivariate analysis, was performed on datasets containing only identified metabolites to further examine the separations between metabolome profiles of the experimental groups and potentially identify patterns associated with them. Figure 4A shows the two-dimensional score plot generated from this analysis. In contrast to the PCoA plot (Figure 3A), greater overlap was observed between LB and CO than with TC. The variable importance in the projection (VIP) scores plot illustrates the metabolites and their contributions to the observed separation in the PLS-DA plot. Figure 4B,C list the top 30 metabolites for components 1 and 2 of the PLS-DA model, along with their respective VIP scores. The relative concentrations for each metabolite in each corresponding group are depicted by the colored boxes on the right. As the model compares all the groups, the metabolites with the highest VIP scores are those that are distinct across each experimental group, as observed with xanthosine (VIP score; Comp. 1 = 3.1; Comp. 2 = 2.8).

**Figure 5:**
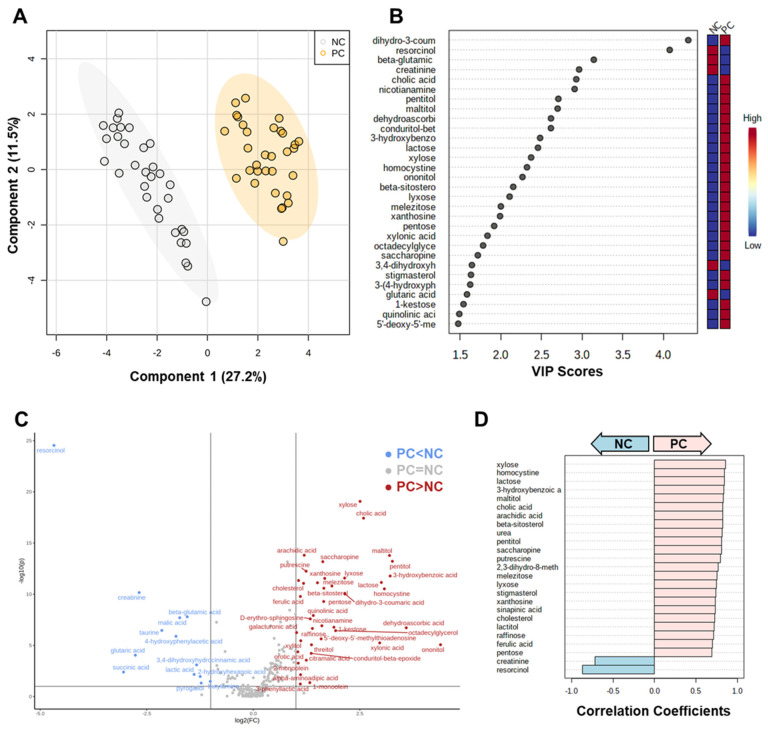
Comparison of only the control groups. (**A**) Partial least squares discriminant analysis (PLS-DA) of only the control groups. The shaded ellipses indicate the 95% confidence regions. (**B**) Top 30 metabolites ranked based on the PLS-DA variable importance in projection (VIP) score for component 1. (**C**) Volcano plot of annotated metabolites between the NC and PC group (important features were selected based on a 2-fold change threshold and an FDR-adjusted *p* < 0.1). Red circles represent significant features that are greater in the PC group; blue circles represent those greater in NC. Features in gray are statistically insignificant to the separation between groups. (**D**) Correlation plot of metabolites significantly associated with a given pattern ‘PC–NC’ using Spearman rank correlation distance measure. Light pink indicating positive correlations with PC and that in light blue indicating negative correlations. Abbreviations: NC—negative control; PC—positive control.

A permutation test was performed based on prediction accuracy and yielded an empirical *p* < 0.001 (0/1000 permutations) for the model. Furthermore, the prediction accuracy of the PLS-DA model and other performance measures were calculated using 10-fold cross-validation (Supplementary Table S2). Based on these calculations, five components are required for optimal performance, achieving a 96% accuracy rate, R^2^ = 0.93, and Q^2^ = 0.86. By contrast, a model created from a subset containing only the control groups yielded a higher accuracy rate, R^2^, and Q^2^ with component 1 (Supplementary Table S2). However, the permutation test yielded an empirical *p* value of 0.157 (157/1000 permutations). This is a common challenge associated with PLS-DA, which is known for its propensity to overfit data due to its consideration of class labels. Interestingly, a similar separation between the control groups was observed on the two-dimensional PLS-DA score plot of the control groups (Figure 5A), as seen on the PCoA plot (Figure 3B). A greater range was also observed among the VIP scores of the top 30 metabolites for this analysis (Figure 5B).

Univariate analysis was performed on the subset containing only the control groups to investigate the significantly different features. Annotated metabolites with a 2-fold change minimum between the groups and an FDR-adjusted *p* < 0.1 are visualized on the volcano plot (Figure 5C). Based on these parameters, 39 metabolites were more abundant in samples of the PC group and 13 in the NC group. The results of the correlation analysis using the Spearman rank correlation distance measure are summarized in Figure 5D. A greater number of compounds had a strong positive correlation with the PC group than with NC.

**Figure 6:**
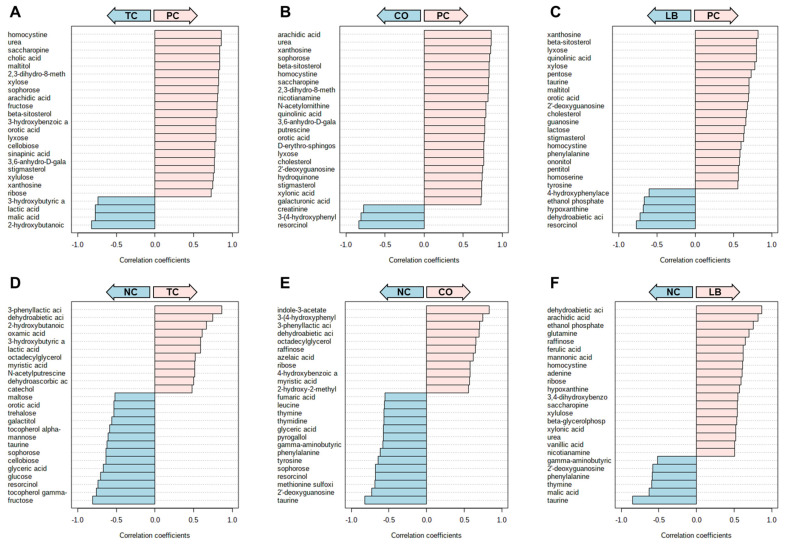
Correlation plots of significant metabolites between the control and treatment groups using Spearman rank correlation distance measure. Groups in light pink arrows are positively correlated with metabolites presented in pink bars. Likewise, groups in light blue are positively correlated with metabolites with light blue bars. (**A**) Correlation plot of metabolites associated with PC or TC groups. (**B**) Correlation plot of metabolites associated with PC or CO groups. (**C**) Correlation plot of metabolites associated with PC or LB groups. (**D**) Correlation plot of metabolites associated with NC or TC groups. (**E**) Correlation plot of metabolites associated with NC or CO groups. (**F**) Correlation plot of metabolites associated with the NC or LB groups. Abbreviations: NC—negative control; TC—*trans*-cinnamaldehyde; LB—*Lactobacillus* strains; CO—combination of TC and LB; PC—positive control.

**Figure 7:**
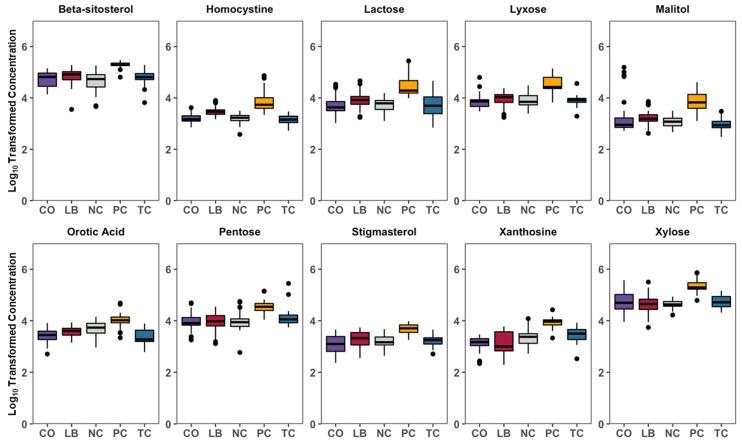
Box plots summarizing the general log-transformed concentration of metabolites that were significantly correlated with PC compared to all other groups. Black spots represent individual samples out of the 32 total per group (8 ceca X 4 replicates). Differences considered significant by Kruskal–Wallis H test by FDR correction at *p*  <  0.05. Abbreviations: NC—negative control; TC—*trans*-cinnamaldehyde; LB—*Lactobacillus* strains; CO—combination of TC and LB; PC—positive control.

**Figure 8:**
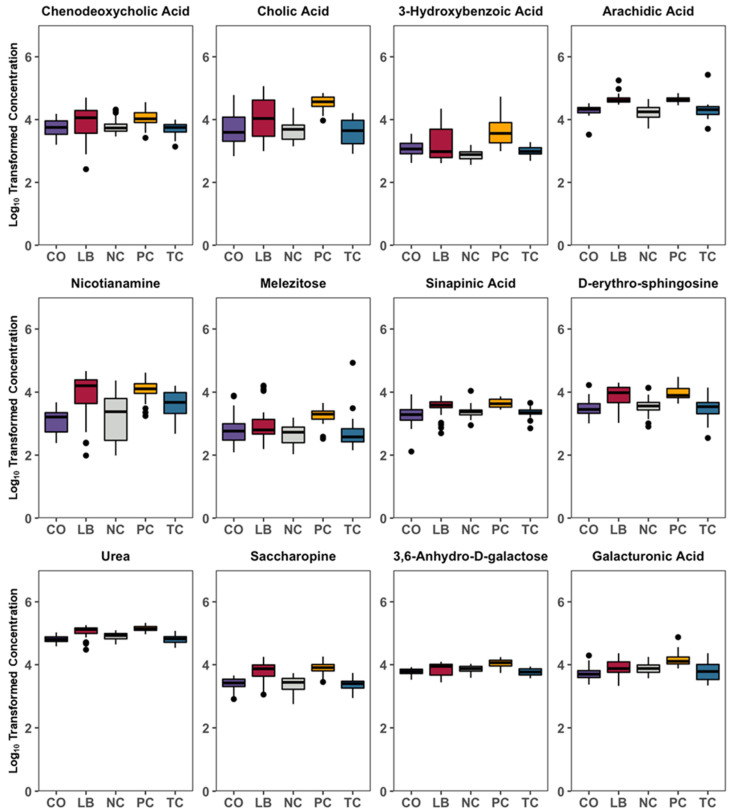
Box plots summarizing the general log-transformed concentration of metabolites that were significantly correlated with PC compared to the NC, TC, and CO groups. Black spots represent individual samples out of the 32 total per group (8 ceca X 4 replicates). Differences considered significant by Kruskal–Wallis H test by FDR correction at *p*  <  0.05. Abbreviations: NC—negative control; TC—*trans*-cinnamaldehyde; LB—*Lactobacillus* strains; CO—combination of TC and LB; PC—positive control.

**Figure 9:**
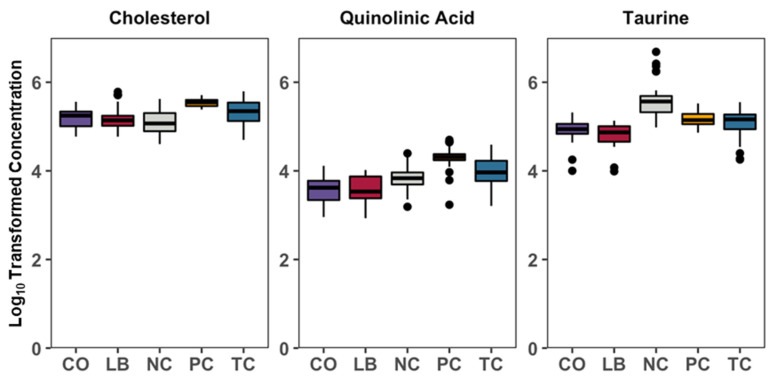
Box plots summarizing the general log-transformed concentration of metabolites that were significantly correlated with PC compared to the NC, LB, and CO groups. Black spots represent individual samples out of the 32 total per group (8 ceca X 4 replicates). Differences considered significant by Kruskal–Wallis H test by FDR correction at *p*  <  0.05. Abbreviations: NC—negative control; TC—*trans*-cinnamaldehyde; LB—*Lactobacillus* strains; CO—combination of TC and LB; PC—positive control.

**Figure 10:**
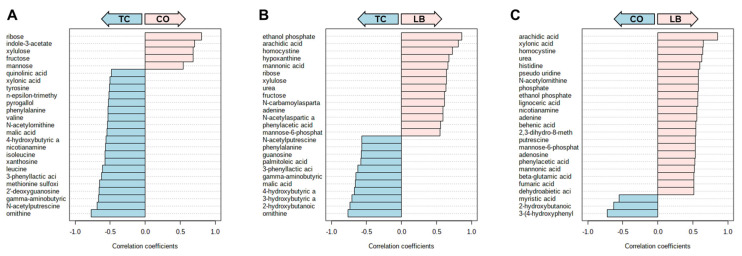
Correlation plots of significant metabolites between the treatment groups using Spearman rank correlation distance measure. Groups in light pink arrows are positively correlated with metabolites presented in pink bars. Likewise, groups in light blue are positively correlated with metabolites, as indicated by the light blue bars. (**A**) Correlation plot of metabolites associated with TC or CO groups. (**B**) Correlation plot of the metabolites associated with the TC or LB groups. (**C**) Correlation plot of metabolites associated with LB or CO groups. Abbreviations: TC—*trans*-cinnamaldehyde; LB—*Lactobacillus* strains; CO—combination of TC and LB.

Correlation analyses of the treatment groups with either the PC (Figure 6A–C) or NC (Figure 6D–F) group are presented in Figure 6. Thirteen compounds are uniformly correlated with PC when compared to all other experimental groups, including beta-sitosterol, stigmasterol, xylose, orotic acid, xanthosine, nicotinamine, pentose, and lactose (Figure 7). Additionally, comparisons with the NC, TC, and CO groups yielded 14 metabolites that were not observed in the LB group. This included chenodeoxycholic acid, cholic acid, urea, saccharopine, arachidic acid, sinapinic acid, and 3-hydroxybenzoic acid (Figure 8). Cholesterol, quinolinic acid, and taurine were observed in the analysis of PC compared to NC, LB, and CO, but not with TC (Figure 9). Several of these metabolites were also observed on plots generated from correlation analysis performed between the treatment groups (Figure 10).

**Figure 11:**
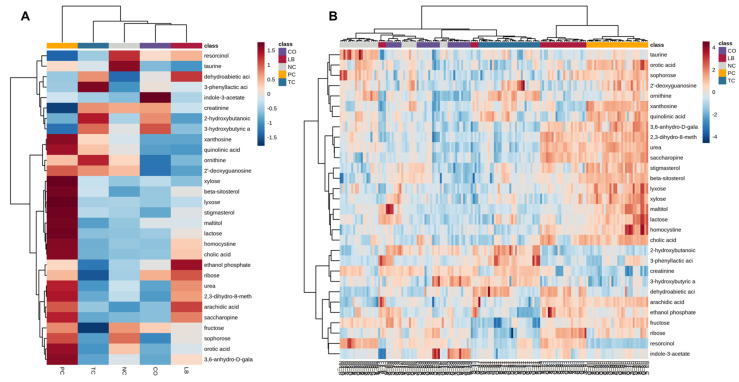
Heatmap representation of the top 30 most significant metabolites that distinguish between experimental groups as determined by Student’s *t*-test. (**A**) Heatmap constructed with hierarchical clustering of group averages. (**B**) Heatmap constructed with hierarchical clustering of individual samples. Abbreviations: NC—negative control; TC—*trans*-cinnamaldehyde; LB—*Lactobacillus* strains; CO—combination of TC and LB; PC—positive control.

The top 30 metabolite concentrations based on Student’s t-test are visualized by heat mapping either using group averages (Figure 11A) or individual samples (Figure 11B). Many top metabolites were more abundant in PC samples, as observed by the greater proportion of red boxes along the columns. This may contribute to the distinct clustering of the PC group samples as observed by the dendrograms drawn along the top. The four replicates of each sample also appear to cluster together in Figure 11B.

**Figure 12:**
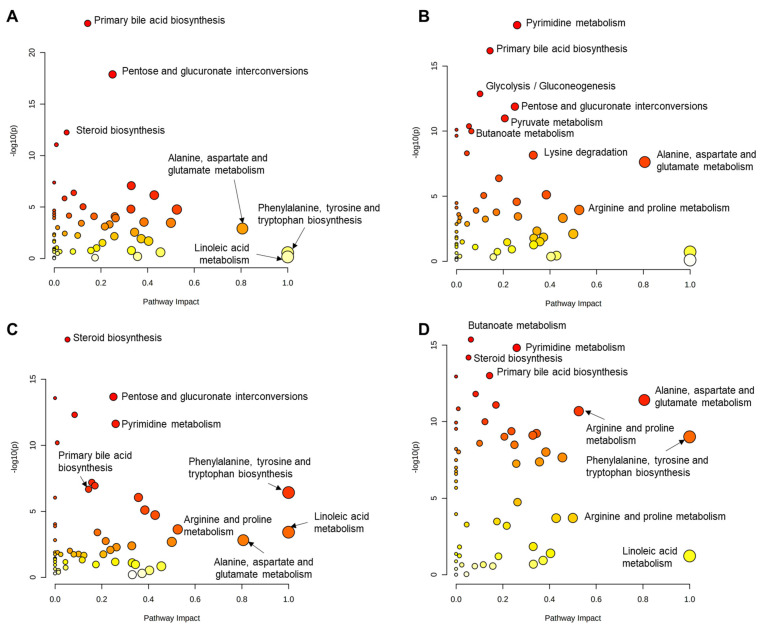
Overview of all metabolic pathways as identified by quantitative pathway enrichment (−log_10_ (*p*)) and topology (pathway impact) analysis, mapped to the *Gallus gallus* (chicken) KEGG pathway library. The vertical axis shows the –log(*p*) value from enrichment analysis, indicating statistical significance, while the horizontal axis represents pathway impact based on topological centrality. Each circle corresponds to a pathway, with size reflecting the centrality of involved metabolites and color intensity (yellow to red) indicating increasing statistical significance. Metabolic pathway analyses performed by pairwise comparison of (**A**) PC vs. NC. (**B**) PC vs. TC. (**C**) PC vs. LB. (**D**) PC vs. CO. Abbreviations: NC—negative control; TC—*trans*-cinnamaldehyde; LB—*Lactobacillus* strains; CO—combination of TC and LB; PC—positive control.

An overview of the pathway analyses comparing the PC group to the NC, TC, LB, or CO groups is illustrated in Figure 12A–D. The top three pathways based solely on enrichment analysis of samples in the NC and PC groups are primary bile acid biosynthesis, pentose and glucuronate interconversions, and steroid biosynthesis (Figure 12A; Supplementary Table S3). Among the pathways with -Log_10_ *p* values > 2, the alanine, aspartate, and glutamate metabolism pathway had the highest impact score. Primary bile acid biosynthesis and steroid biosynthesis were also ranked highly in the enrichment analysis conducted for the treatment groups. However, pyrimidine metabolism was the top pathway for TC (Figure 12B) and LB (Figure 12C), and second to butanoate metabolism in the CO group (Figure 12D). As observed with NC, alanine, aspartate, and glutamate metabolism, and arginine and proline metabolism ranked highly among all treatment groups based on topology analyses. The phenylalanine, tyrosine, and tryptophan biosynthesis pathway had the greatest impact score, but its significance was only evident in comparisons of PC with the LB (Figure 12C) and CO (Figure 12D) groups.

**Figure 13:**
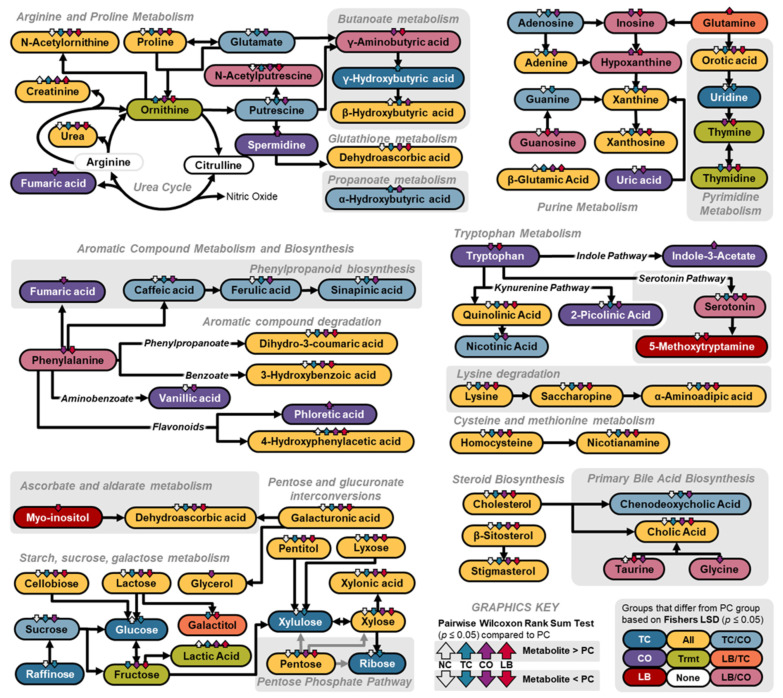
Schematic diagram illustrating the cecal metabolites and their corresponding pathways, as detected by gas chromatography–Time-of-Flight mass spectrometry. The color of the text boxes represents the treatments that exhibited significant differences compared to the PC group as determined by a one-way ANOVA using Fisher’s LSD post hoc test (*p*  <  0.05). The arrows indicate the pairwise differences between the corresponding groups, compared to the PC group using the Wilcoxon rank sum test (*p*  <  0.05). The direction of the arrow indicates the relative abundance of the metabolite in the group, relative to its presence in the PC group. Abbreviations: NC—negative control; TC—*trans*-cinnamaldehyde; LB—*Lactobacillus* strains; CO—combination of TC and LB; PC—positive control.

A summary of the individual metabolites that are statistically different from the PC group based on Fisher’s LSD and pairwise Wilcoxon rank sum test (*p* < 0.05) and their relation to the pathways is summarized in Figure 13. Derivatives of cinnamaldehyde, such as 3,4-dihydroxycinnamic acid (caffeic acid), 4-hydroxy-3-methoxycinnamic acid (ferulic acid), and 3,5-dimethoxy-4-hydroxy-cinnamic acid (sinapinic acid), were lower in the NC, TC, and CO groups compared to the PC.

## 4. Discussion

Reducing *Salmonella* colonization in the gastrointestinal tract of turkeys is crucial, as it can help mitigate the incidence of salmonellosis, particularly given the significant role that poultry products play in the transmission of the pathogen. Preventing *Salmonella* colonization most likely involves comprehensive interactions between the treatments and their impact on the poult, intestinal microbes, and the metabolites they produce [25]. The newly hatched poults are more susceptible to colonization by *Salmonella* as they lack the protection conferred by well-developed microflora within their gastrointestinal tract. The current study analyzes changes to the cecal metabolome in response to *S.* Heidelberg colonization and how the application of treatments affects these alterations. The ceca are particularly interesting as they host abundant bacterial communities, including pathogens such as *Salmonella*. This is partly due to the slow passage of digesta, which offers an ideal environment for the microbes [26,27,28,29]. Furthermore, the ceca serve important functionality in water and electrolyte absorption, making it an ideal segment to evaluate the response to the *S.* Heidelberg challenge and the treatments applied [30].

The distinct and more convergent cecal metabolome profiles of samples in the PC group suggest that the alterations may be primarily associated with *S.* Heidelberg colonization (Figure 3). Among the metabolites correlated with the PC group were several sugar moieties, including lactose, pentose, xylose, lyxose, and cellobiose, as well as several sugar alcohols, which were present at greater abundances than in the other groups (Figure 7). Similarly elevated levels of lactose had also been reported among *S.* Typhimurium-infected broiler chicks and mice [31,32]. Likewise, mice fed lactose- and cellobiose-rich diets were found to harbor increased loads of *S.* Typhimurium in fecal and cecal contents than their counterparts fed the conventional diet (Kim et al., 2017) [15]. *Campylobacter jejuni* colonization was also found to reduce glucose uptake in the jejunum of infected birds [33]. Elevated levels of raffinose, sucrose, fructose, and sugar alcohols in the cecal contents of mice that were treated with antibiotics were also correlated with increased susceptibility to *C. difficile* infection [34]. Thus, the accumulation of sugars that are typically metabolized by commensal microbes suggests that *S.* Heidelberg may either impair their absorption or alter the microbial population of the ceca.

Primary bile acid biosynthesis was notably affected, with several metabolites associated with this pathway detected at higher levels in the PC group compared to the NC group (Figure 13). Similar findings were observed in fecal samples of *Salmonella*-infected mice, though the mechanisms behind the increase in primary bile acids with *Salmonella* infections remain unclear [35]. Certain strains of *L. salivarius* were reported to exhibit bile salt hydrolase (BSH) activity, albeit at very low levels [36]. This may contribute to the observed differences in taurine and glycine presence between the LB and CO groups and PC (Figure 13), as the deconjugation catalyzed by BSH liberates the glycine and taurine moieties from the steroid core. Host physiology may be influenced by microbial BSH due to the function of bile acids in facilitating lipid absorption, in cholesterol homeostasis, acting as signaling molecules regulating their biosynthesis, and as part of the mucosal defense in the intestine [37,38]. The liberated amino acids from bile salt deconjugation can potentially be utilized as carbon, nitrogen, and energy sources for bacteria. However, lactobacilli were not shown to use the steroid moiety of the bile salt, though they likely use the amino acid portion [39].

By forming biofilms, *Salmonella* has adapted to resist high levels of bile in environments such as the gallbladder [40,41]. Studies on *S.* Typhi observed that the upregulation of the O-antigen capsule during growth in bile plays a crucial role in biofilm formation on gallstones and cholesterol-coated surfaces [42]. Both are important to the asymptomatic carriage of the pathogen. Bile was also reported to downregulate the expression of genes involved in motility and cell invasion, which are encoded on the *Salmonella* pathogenicity island 1 (SPI-1) [43,44]. Specifically, chenodeoxycholic acid demonstrated the greatest anti-infective activity against *S.* Typhimurium ex vivo, inhibiting its ability to invade cells by repressing expression of the SPI-1 virulence gene [45]. Although these impact their capacity for intestinal invasion, they would not necessarily affect their ability for luminal expansion. Similarly, cholic acid supplementation altered the composition of the cecal microbiota, preferentially regulating the growth of certain bacteria [46]. Higher levels of chenodeoxycholic acid and cholic acid observed in the PC group may confer a survival advantage to the pathogen in the cecal lumen (Figure 8).

Depletion of amino acids by the microbiota would exert selective pressure on microbes that require amino acid biosynthesis pathways, forcing pathogens such as *S.* Typhimurium to maintain prototrophic growth [47]. Among the differences observed between the control and treatment groups is the wide array of metabolites generated through amino acid metabolism. In this study, treatment groups and the NC had a lower abundance of amino acids compared to the PC group (Figure 13). Metabolites associated with arginine and proline metabolism were present at higher abundances in the PC group compared to the treated groups, consistent with findings from studies investigating *Salmonella* infection in chickens [48,49]. L-Arginine is an essential amino acid for birds. Its conversion into urea and L-ornithine is catalyzed by arginase, while nitric oxide synthase (NOS) catalyzes the transformation into nitric oxide and L-citrulline [50]. Nitric oxide is an effective antimicrobial agent and plays a central role in innate immunity and regulation of immune function, particularly against intracellular pathogens such as *Salmonella* [51,52]. Thus, channeling arginine availability towards reaction with arginase would likely suppress nitric oxide production, potentially providing an advantage for *Salmonella* colonization in the gut. The intermediate in the methionine biosynthetic pathway, homocysteine, has demonstrated the ability to enhance *Salmonella* resistance to s-nitrosothiols and contribute to its defenses against nitrosative stress in murine salmonellosis [53]. Similarly, greater levels of homocysteine were observed in poults challenged with *S.* Heidelberg and not treated with any interventions (Figure 7).

The degradation of aromatic amino acids by proteolytic bacteria generates a variety of phenolic and indolic compounds. Tryptophan metabolites like indole and kynurenine are known to bind to aryl hydrocarbon receptor (AhR), a component involved in the innate immune response, and contribute to enhancing intestinal barrier integrity. Intestinal microorganisms can transform tryptophan into mono-substituted indole compounds, such as indole-3-acetic acid, which serve as ligands for the AhR. The targeted metabolomic analysis found that indole derivatives generated through tryptophan metabolism by *Lactobacillus* species hindered colonization of pathogenic *Candida albicans* through AhR-dependent expression of IL-22, which stimulates epithelial cells to produce antimicrobial proteins [54]. However, *S.* Typhimurium can subvert the IL-22-dependent host-defense mechanism by acquiring iron through the siderophore salmochelin. Thus, this immune reaction may enhance *Salmonella* colonization by starving susceptible microbes and thereby eliminating competition with which the pathogen must contend [55]. Alternately, decreased levels of tryptophan and indole-3-acetic acid have been observed in the fecal samples of individuals with inflammatory bowel disease. This may be alleviated by probiotic *Lactobacillus reuteri*, which produces indole derivatives from dietary tryptophan [56,57].

Several of the metabolites were also more strongly associated with certain treatments. Except for γ-aminobutyric acid (GABA), most hydroxybutyric acids were notably higher in the groups receiving cinnamaldehyde (Figure 13). Antibiotic-mediated depletion of anaerobes reduces butyrate levels, promoting the expansion of *Salmonella* in the gut lumen [58]. β-hydroxybutyric acid is a ketone body that serves as a nutrient source enhanced by the gut microbiota during fasting [59]. Antibiotic treatment in mice has led to decreased cecal GABA levels, suggesting that the microbiota contributed to its production [60]. Accumulation of the monoamine neurotransmitter serotonin in the intestinal lumen was shown to downregulate the expression of virulence genes in *C. rodentium*, decreasing the host’s susceptibility to the pathogen. *Salmonella* also encodes the same serotonin receptor and may also be affected [61]. Its role in inflammation within the gut has been demonstrated through its observed influence on proinflammatory cytokine production during experimentally induced colitis [62,63,64]. Increased serotonin levels in the cecal contents of birds in the PC group were also observed in this study (Figure 13). The presence of *S.* Heidelberg may similarly stimulate the release of serotonin, as was observed with *Escherichia coli* lipopolysaccharide [65].

Additionally, Yang and colleagues demonstrated the necessity of de novo pyrimidine synthesis for *S.* Typhimurium colonization of chicks [66]. Specifically, they noted a decrease in the colonization of chicks with mutants lacking the *pyrE* gene, which encodes orotate phosphoribosyltransferase. This enzyme catalyzes the formation of orotidine 5′-monophosphate from orotic acid, which was present at greater levels in the PC group (Figure 7) [67]. Although the necessary colonization factors often differ based on the host, purine and pyrimidine metabolism genes were among the few that were essential for *S.* Typhimurium colonization across multiple food-producing animals [68].

Differences and similarities were also observed among the treatment groups, with a greater proportion of individual metabolites and pathways being more similar between the TC and CO groups than between the LB and CO groups. The greater spread observed within the LB group may be a contributing factor to these differences. Yet, the divergence between treatments was not as pronounced as that of the PC group. In vitro investigations simulating fermentation in the gut of pigs demonstrated that the antimicrobial effect of *trans*-cinnamaldehyde may be species-specific, as coliforms were more strongly affected than lactic acid bacteria [69]. The specificity may favor lactic acid-producing bacteria, as lactic acid was a metabolite found at higher concentrations in the treatment groups compared to the PC. Conversely, lactic acid was found in higher concentrations in the treatment groups compared to the untreated challenged birds (Figure 6D, Figure 13). Lactic and malic acid are known to destabilize and disintegrate the outer membrane of *Salmonella* [70]. Groups that were fed cinnamaldehyde had lower concentrations of cinnamaldehyde derivatives, including caffeic acid, ferulic acid, and sinapinic acid, compared to the PC group, but this was not observed in the group that received only the *Lactobacillus* cocktail (Figure 13).

The *Lactobacillus* species, which generally maintains dominance within the avian gastrointestinal tract, is often described to exhibit low abundance in the cecum. However, the heterogeneity of the genus yields an observable temporal variation in the *Lactobacillus* species present. Despite the dynamic fluctuations in their abundance with age and diet, studies have consistently observed a notable presence of *L. salivarius* across different segments, including the ceca [71,72]. This was not the same for *L. ingluviei.* Thus, future investigations with only the use of *L. salivarius* may be warranted. A marked increase in *L. salivarius* abundance has been observed in chicken ilea after day 15 [71].

Studies in mice have shown that the similarity of the resident microbes to the incoming species influences the ability of the microbes to colonize within the gut. Stetcher and colleagues observed more efficient colonization of a commensal *L. reuteri* strain when high populations of Lactobacilli were present, whereas a greater population of commensal *E. coli* led to increased susceptibility to *Salmonella* colonization [73]. The microbiota was found to confer resistance to pathogen colonization and mediate their clearance following infection [74]. Thus, understanding the impact of the interventions on the cecum would provide additional insights into better strategies to mitigate the risk of *Salmonella* carriage and shedding.

Taken together, these findings suggest that the *S.* Heidelberg challenge affected the cecal metabolome to a greater extent than the application of the treatments. We speculate that the changes in the relative abundance of metabolites among the groups are associated with the observed reduction in *S.* Heidelberg recovered from the ceca [18]. One of the strengths of the present study is the replicate analysis of individual samples, which allowed for consideration of variability during data acquisition. It represents the early dissection of the complex relationship between metabolite changes associated with the pathogen, microbiota, host, and treatments. Although the differences observed were significant enough to be detected, the possibility of external factors influencing the changes to the metabolome outside of the *S.* Heidelberg challenge and treatment with *trans*-cinnamaldehyde or lactobacilli still exists. Therefore, further investigation is necessary to establish a definitive causal relationship between the observed shifts in metabolites and either *S.* Heidelberg colonization or the application of treatments, while ruling out external factors that may have influenced the outcomes. The use of targeted metabolomics and in vitro assays provides a better understanding of the mechanisms underlying *S.* Heidelberg’s persistence and validates the effectiveness of interventions. Additionally, conducting further analyses to identify unannotated metabolites may yield valuable information about the observed changes.

## 5. Conclusions

The metabolomic analysis conducted in this study revealed changes in cecum-associated metabolites that are likely a response to the presence of *S.* Heidelberg in challenged birds and the application of the *trans*-cinnamaldehyde or *Lactobacillus* treatments. The impact of *S.* Heidelberg was tangible, as the poults in the challenged and untreated group had the most distinct metabolome profiles, diverging the furthest from the unchallenged control. The accumulation of metabolites, such as sugars (lactose) and primary bile acids (chenodeoxycholic acid and cholic acid), in the challenged control could confer an advantage for *S.* Heidelberg’s luminal expansion. It may be indicative of shifts in the microbiota. Conversely, metabolites known to disrupt *Salmonella* colonization, such as lactic acid, were present in greater abundance in the unchallenged and treated groups. These findings serve as a basis for further studies that investigate the potential causal relationship between the observed shifts and *S.* Heidelberg colonization in the ceca. Understanding these alterations can help elucidate the modifications occurring in the intestinal microenvironment, which facilitate *Salmonella’s* persistence in the avian cecum. This, in turn, can be used to better implement pre-harvest interventions, thereby optimizing their efficacy in reducing *Salmonella* within the flock. The findings are also expected to provide valuable insights into the reduction in *S.* Heidelberg achieved by the interventions and elucidate ways to optimize them for pre-harvest applications in adult turkeys.

## Data Availability

The original contributions presented in this study are included in the article. Further inquiries can be directed to the corresponding author.

## Supplementary Materials

The following supporting information can be downloaded at https://www.mdpi.com/article/10.3390/ani15142016/s1, Table S1: Results of PERMANOVA and ANOSIM tests using Euclidean distances metric of cecal metabolome between experimental groups (permutation = 999). NC—negative control, TC—*trans*-cinnamaldehyde, LB—*Lactobacillus* strains, CO—combination of TC and LB, PC—positive control.; Table S2: PLS-DA performance measures measured by 10-fold cross validation with reported accuracy rate, R^2^ and Q^2^ value for five components. NC—negative control, TC—*trans*-cinnamaldehyde, LB—*Lactobacillus* strains, CO—combination of TC and LB, PC—positive control.; Table S3: Significant pathways (−log *p*s > 2) between PC and other experimental groups as identified by quantitative pathway enrichment and topology analysis conducted using the *Gallus gallus* (chicken) KEGG library. NC—negative control, TC—*trans*-cinnamaldehyde, LB—*Lactobacillus* strains, CO—combination of TC and LB, PC—positive control.
