# Supplementary material for: Cecal Metabolome Profiles of Turkey Poults in Response to Salmonella Heidelberg Challenge with or Without Turkey-Derived Lactobacillus Probiotic and Trans-Cinnamaldehyde
Source: Animals (Basel). 2025 Jul 8;15(14):2016. doi: 10.3390/ani15142016 (PMC12291739; doi:10.3390/ani15142016)
Supplement: Supplementary file 1 [file animals-15-02016-s001.zip › animals-3699198-supplementary.pdf]

SUPPLEMENTARY MATERIALS

Supplementary Table S1. Results of PERMANOVA and ANOSIM tests using Euclidean distances metric of cecal metabolome between experimental groups (permutation = 999). NC – negative control, TC – *trans*-cinnamaldehyde, LB – *Lactobacillus* strains, CO – combination of TC and LB, PC – positive control.

Comparison		PERMANOVA		ANOSIM	
		R^2	*p*-value	R Statistic	*p*-value
All Groups		0.242	0.001	0.500	0.001
CO	LB	0.103	0.001	0.322	0.001
CO	TC	0.064	0.001	0.153	0.001
LB	TC	0.123	0.001	0.391	0.001
NC	LB	0.125	0.001	0.448	0.001
NC	CO	0.125	0.001	0.378	0.001
NC	TC	0.100	0.001	0.312	0.001
PC	NC	0.305	0.001	0.869	0.001
PC	TC	0.261	0.001	0.779	0.001
PC	CO	0.254	0.001	0.709	0.001
PC	LB	0.186	0.001	0.554	0.001

Supplementary Table S2. PLS-DA performance measures measured by 10-fold cross validation with reported accuracy rate, R^2 and Q^2 value for five components. NC – negative control, TC – *trans*-cinnamaldehyde, LB – *Lactobacillus* strains, CO – combination of TC and LB, PC – positive control.

Groups in Model	Measure	1 comps	2 comps	3 comps	4 comps	5 comps
All (NC, PC, LB, CO, TC)	Accuracy	0.344	0.617	0.869	0.921	0.958
	R^2	0.578	0.741	0.853	0.901	0.930
	Q^2	0.501	0.677	0.795	0.834	0.861
Controls (NC & PC)	Accuracy	0.980	1.000	1.000	1.000	1.000
	R^2	0.850	0.938	0.976	0.986	0.992
	Q^2	0.830	0.915	0.947	0.959	0.962

Supplementary Table S3. Significant pathways (-log *ps* > 2) between PC and other experimental groups as identified by quantitative pathway enrichment and topology analysis conducted using the *Gallus gallus* (chicken) KEGG library.NC – negative control, TC – *trans*-cinnamaldehyde, LB – *Lactobacillus* strains, CO – combination of TC and LB, PC – positive control.

Pathway	Impact	NC vs PC			TC vs PC			LB vs PC			CO vs PC		
		Raw p	- Log_{10} (p)	FDR	Raw p	- Log_{10} (p)	FDR	Raw p	- Log_{10} (p)	FDR	Raw p	- Log_{10} (p)	FDR
Alanine, aspartate and glutamate metabolism	0.806	0.001	2.91	0.002	0.000	7.63	0.000	0.002	2.82	0.004	0.000	11.41	0.000
Arginine and proline metabolism	0.525	0.000	4.76	0.000	0.000	3.95	0.000	0.000	3.64	0.001	0.000	10.68	0.000
D-Glutamine and D-glutamate metabolism	0.500	0.000	3.47	0.001	0.008	2.11	0.013	0.002	2.69	0.005	0.000	3.71	0.000
Arginine biosynthesis	0.385	0.000	3.54	0.001	0.000	5.11	0.000	0.000	5.10	0.000	0.000	8.02	0.000
Lysine degradation	0.329	0.000	4.80	0.000	0.000	8.16	0.000	0.004	2.39	0.010	0.000	9.10	0.000
Cysteine and methionine metabolism	0.263	0.000	3.92	0.000	0.000	3.45	0.001	0.005	2.29	0.012	0.000	4.75	0.000
Pyrimidine metabolism	0.260	0.000	3.97	0.000	0.000	17.92	0.000	0.000	23.62	0.000	0.000	15.08	0.000
Pentose and glucuronate interconversions	0.250	0.000	18.14	0.000	0.000	13.79	0.000	0.000	13.07	0.000	0.000	7.44	0.000
Pyruvate metabolism	0.207	0.018	1.73	0.028	0.000	11.58	0.000	0.019	1.72	0.032	0.000	9.25	0.000
Glycolysis / Gluconeogenesis	0.206	0.003	2.46	0.007	0.000	13.46	0.000	0.018	1.73	0.032	0.000	8.82	0.000
Tyrosine metabolism	0.171	0.000	4.10	0.000	0.000	3.77	0.000	0.000	6.95	0.000	0.000	11.09	0.000
Purine metabolism	0.145	0.000	6.38	0.000	0.000	3.75	0.000	0.000	11.98	0.000	0.000	11.36	0.000
Primary bile acid biosynthesis	0.144	0.000	22.84	0.000	0.000	16.17	0.000	0.000	6.68	0.000	0.000	13.01	0.000
Glutathione metabolism	0.124	0.000	5.03	0.000	0.001	3.24	0.001	0.021	1.67	0.034	0.000	9.99	0.000
Butanoate metabolism	0.064	0.000	4.17	0.000	0.000	9.99	0.000	0.010	2.02	0.020	0.000	15.37	0.000
Steroid biosynthesis	0.053	0.000	12.25	0.000	0.000	10.38	0.000	0.000	18.01	0.000	0.000	14.19	0.000
Steroid hormone biosynthesis	0.009	0.000	11.06	0.000	0.000	3.60	0.001	0.000	10.19	0.000	0.000	10.84	0.000
Phenylalanine, tyrosine and tryptophan biosynthesis	1.000	0.270	0.57	0.302	0.189	0.72	0.225	0.000	6.43	0.000	0.000	9.01	0.000
